# Cortical Vein Thrombosis after Infliximab Treatment for Crohn’s Disease

**DOI:** 10.3390/neurolint13010011

**Published:** 2021-03-11

**Authors:** Saeed Razmeh, Nafiseh Niknam, Negar Sadat Rabbani, Shekoofeh Nikoee, Fatemeh Vafa Pour, Laleh Taghavian

**Affiliations:** 1 Department of Neurology, Shahid Beheshti Hospital, Yasuj University of Medical Sciences, Yasuj 75914-93686, Iran; 2 Student Research Committee, Yasuj University of Medical Sciences, Yasuj 75914-93686, Iran; niknamnafiseh@gmail.com (N.N.); negarsadat.rabani@gmail.com (N.S.R.); shekoofeh-nikooei@yums.ac.ir (S.N.); 3 Department of Internal Medicine, Emam Sajad Hospital, Yasuj University of Medical Sciences, Yasuj 75914-93686, Iran; vafapoorfatemfaeh@gmail.com; 4 Nursing Department, Shahid Beheshti Hospital, Yasuj University of Medical Sciences, Yasuj 75914-93686, Iran; lalehtaghavian842@gmail.com

**Keywords:** cortical vein thrombosis, infliximab, Crohn’s disease

## Abstract

Inflammatory bowel disease (IBD) including ulcerative colitis and Crohn’s disease puts patients at high risk of thromboembolism accidents. These patients may take infliximab for active and fistulating Crohn’s disease, which can also increase the risk of thrombosis. Deep vein thrombosis (DVT) and pulmonary thromboembolism (PTE) are more common among these patients, but cerebrovascular, mesenteric, portal and retinal veins can also be affected. In this paper, we report a case of isolated right Labbe vein thrombosis after infliximab therapy for Crohn’s disease. To the best of our knowledge, our patient is the first case report of isolated cortical vein thrombosis following administration of rituximab for Crohn’s disease.

## 1. Introduction

Cortical venous thromboses are mostly caused by sinus thromboses which constitute a small portion of all types of strokes. Isolated cortical venous thromboses (ICVTs) are much rarer and only have been reported in case reports [1,2]. The vein of Labbe, also known as the inferior anastomotic vein, is one of the cortical veins that anastomoses between the middle cerebral vein and transverse sinus and drains the lateral temporal lobe’s blood [3]. Risk factors for cerebral vein thrombosis include trauma, inherited and acquired thrombophilia, malignancy, exogenous hormones and pregnancy [2,4].

Crohn’s disease is a type of inflammatory bowel disease (IBD), and its symptoms are very diverse and mainly affect the gastrointestinal system [5]. The inflammatory state of the disease can cause an increased level of cytokines, damaging endothelium, activating immune cells and enhancing coagulation cascade [6], and it also puts patients at high risk of thromboembolism accidents [7]; therefore in a hospitalized IBD patient with active disease, anticoagulants are recommended to prevent such accidents as deep vein thrombosis, pulmonary thromboembolism, etc. [8].

Infliximab is a monoclonal antibody that is used in the active form of Crohn’s disease and treatment of fistulas in this disease. Studies have shown that there is an association between treatment with infliximab and vascular thrombosis, but that is very rare [9].

Here we report on a known case of Crohn’s disease, presented with a chief complaint of severe headache after infliximab therapy and diagnosed with the vein of Labbe thrombosis.

## 2. Case Presentation

The patient is a 25-year-old female who was admitted to the hospital due to a severe bilateral compressive headache which had started a month prior to admission with a change in patterns like an increase in severity and frequency. She had visited clinics and got painkillers three to four times per week the previous month. She had also experienced diplopia two weeks prior to admission.

Regarding her past medical history, she had Crohn’s disease and several episodes of fistula formation which had been repaired by surgery, and she had received two doses of infliximab three months and one month before admission. Her vitals were all within normal limits. In the physical exam, all components of head and neck, chest, heart and lung sounds, abdomen and extremities were all normal. In the neurological examination, the eye examination revealed left eye abduction limitation and a 4+ papilledema and optic disc hemorrhage in the funduscopic examination; the rest of the neurological exam was intact, including normal mental status, motor, sensory and coordination.

**Figure 1:**
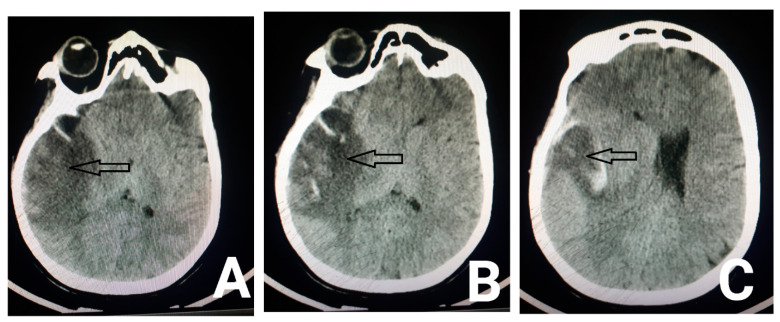
Axial brain CT scan shows hypodensity in the right temporal lobe (arrows in Figure **A**,**B**) in the drainage territory of the vein of Labbe, indicating venous infarct with parenchymal hemorrhage (arrow in figure (**C**)).

**Figure 2:**
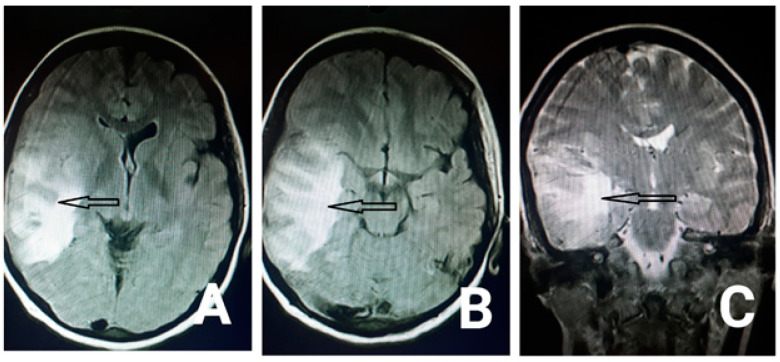
Axial fluid-attenuated inversion (flair) MRI (the arrows in the Figure **A**,**B**) and coronal T2 weighted image (the arrow in the Figure (**C**)) show hyperintensity in the right temporal lobe in the drainage territory of the vein of Labbe.

In terms of her lab data, biochemistry, Prothrombin Time (PT) and Partial Thromboplastin Time (*PTT*) were all within the normal range. Regarding her complete blood count, white blood cell count was 8900/dL, red blood cell count was 3,830,000/dL, hemoglobin was 7 g/dL, hematocrit was 27%, mean corpuscular volume was 70.5 FL, platelet count was 122,000, and ferritin level was 5 ng/mL. Other systemic evaluations such as liver function tests, thyroid function tests, blood urea nitrogen and creatinine were all within the normal range. The first brain CT was normal. The diagnostic and therapeutic lumbar puncture had been done as well. The cerebrospinal fluid (CSF) pressure was 22 cm H2O, and the CSF analysis was normal. We started on acetazolamide (250 milligrams, three times a day) and topiramate (20 milligrams, twice a day); iron and folic acid were added as well. On the third day of hospitalization, the patient developed a decreased level of consciousness and left side hemiparesis, and she was transferred to the ICU and got intubated. Another computed tomography (CT) and magnetic resonance imaging were requested which showed hyperintensity in the right hemisphere and Labbe vein drainage zone, indicating cortical venous thrombosis, although transverse and sagittal sinuses were open (Figure 1 and Figure 2). We started heparin for the patient. She also received two units of packed cells due to her low hemoglobin. Her level of consciousness increased after three days, and she got extubated. All the other symptoms started to resolve, and she was discharged after a week.

## 3. Discussion

Inflammatory bowel disease (IBD) includes ulcerative colitis and Crohn’s disease. The balance between procoagulant and anticoagulant activity in these diseases can be disturbed, and the literature shows that the risk of thrombosis in people with these complications is three to four times higher than those who do not have inflammatory bowel disease. Moreover, thrombosis is more common in ulcerative colitis than Crohn’s disease. It has also been suggested that the inflammation activity and extension of mucosal damage can be associated with the risk of thrombosis [10]. The main pathophysiology for IBD-related thrombosis is not yet demonstrated, however, transient coagulant abnormalities like thrombocytosis, increase in fibrinogen and factor V and VII, antithrombin III and lack of free protein S and C have been suggested to play a role in its mechanism [10]. Jennifer Maag et al. reported superior sagittal sinus thrombosis in a 30-year-old man with Crohn’s disease and factor V Leiden mutation [11]. Deep vein thrombosis and pulmonary thromboembolism (PTE) have been reported to occur more frequently in patients with IBD based on a cohort study, but the cerebrovascular, mesenteric, portal and retinal veins can also be affected [12,13]. Intravenous thrombosis usually occurs in the acute phase of the disease, but Yerby MS et al. reported a case of ulcerative colitis with sagittal sinus thrombosis 10 years after panproctocolectomy. The clinical presentations of cerebral vein thrombosis are variable and depend on factors such as location and extent of thrombosis, patient age and underlying disease. These signs and symptoms include headache, vomiting, seizures, altered consciousness, focal neurological deficit and decreased vision [14]. The importance of cortical venous thrombosis in these patients is because of probable intra cranial hemorrhage, increase in the intracranial pressure, parenchymal lesions, etc. Moreover, these complications caused by Cerebral venous thrombosis may be irreversible and affect patients for the rest of their lives [2].

Infliximab is a kind of monoclonal antibody that binds to tumor necrosis factor-α and is mostly used to reduce inflammatory factors [15]. It is also prescribed for active and fistulating Crohn’s disease [16,17,18]. Besides its beneficial effects like immunomodulation and reduced inflammation, some adverse effects such as an increased risk of thromboembolism have been reported in some cases [5,19,20]. L. GRANGE et al. reported a 31-year-old with refractory ankylosing spondylitis (axial and peripheral involvement) who developed cerebral vein thrombosis after infliximab [21]. Srinivas Puli et al. also reported a case of retinal vein thrombosis after infliximab treatment in a patient with Crohn’s disease [22]. Signs and symptoms of increased intracranial pressure in our patient started one month after the first dose of infliximab. Although the association between infliximab and vascular thrombosis has been identified, its underlying pathophysiology is unknown; various factors appear to be involved in the pathogenesis of thrombosis, and further studies are needed to determine the mechanism of this drug in causing vascular thrombosis. Kolarz Bogdan et al. observed a significant increase in Beta-2-glycoprotein I (B2GPI)–IgM-positive patients during a six-month treatment with infliximab in patients with refractory rheumatoid arthritis (RA) [23].

The most frequently involved sinuses in Cerebral venous thrombosis are the superior sagittal sinus and lateral sinuses. Isolated cortical thrombosis is also rare among Cerebral venous thrombosis, and that is what we deal with in this case. Surprisingly, Labbe vein thrombosis, and especially on the right side, is the least common type of isolated cortical thrombosis [24,25,26].

As we mentioned already, we had a patient with a history of Crohn’s disease, several surgeries and infliximab intake after surgery, who came up with headache, diplopia, eye movement limitation, hemiparesis and decreased level of consciousness and was diagnosed with isolated right Labbe vein thrombosis which could be a rare complication of Crohn’s disease or infliximab. 

## Data Availability

No new data were created or analyzed in this study. Data sharing is not applicable to this article.
